# Physiological Role of Orexinergic System for Health

**DOI:** 10.3390/ijerph19148353

**Published:** 2022-07-08

**Authors:** Ines Villano, Marco La Marra, Girolamo Di Maio, Vincenzo Monda, Sergio Chieffi, Ezia Guatteo, Giovanni Messina, Fiorenzo Moscatelli, Marcellino Monda, Antonietta Messina

**Affiliations:** 1 Department of Experimental Medicine, University of Campania “Luigi Vanvitelli”, 80138 Naples, Italy; marco.lamarra@unicampania.it (M.L.M.); girolamo.dimaio@unicampania.it (G.D.M.); sergio.chieffi@unicampania.it (S.C.); marcellino.monda@unicampania.it (M.M.); antonietta.messina@unicampania.it (A.M.); 2 Department of Movement Sciences and Wellbeing, University of Naples “Parthenope”, 80138 Naples, Italy; vincenzo.monda@uniparthenope.it (V.M.); ezia.guatteo@uniparthenope.it (E.G.); 3 Department of Clinical and Experimental Medicine, University of Foggia, 71100 Foggia, Italy; giovanni.messina@unifg.it (G.M.); fiorenzo400@gmail.com (F.M.)

**Keywords:** orexin/hypocretin, physical activity, obesity, metabolism, neuroprotection, wakefulness, feeding behaviors, energy expenditure

## Abstract

Orexins, or hypocretins, are excitatory neuropeptides involved in the regulation of feeding behavior and the sleep and wakefulness states. Since their discovery, several lines of evidence have highlighted that orexin neurons regulate a great range of physiological functions, giving it the definition of a multitasking system. In the present review, we firstly describe the mechanisms underlining the orexin system and their interactions with the central nervous system (CNS). Then, the system’s involvement in goal-directed behaviors, sleep/wakefulness state regulation, feeding behavior and energy homeostasis, reward system, and aging and neurodegenerative diseases are described. Advanced evidence suggests that the orexin system is crucial for regulating many physiological functions and could represent a promising target for therapeutical approaches to obesity, drug addiction, and emotional stress.

## 1. Introduction

Orexins, or hypocretins, are excitatory neuropeptides that were discovered in the late 1990s, independently and simultaneously by two research groups. The Sakurai group (1998) identified these new neuropeptides using chromatography and named them “orexins” (OrxA and OrxB), from the Greek word “orexis”, meaning “appetite”, due to their ability to stimulate food intake and control the metabolism [1,2]. The other group, de Lecea and colleagues (1998), identified these new neuropeptides using molecular biological techniques and named them hypocretins (Hcrt1 and Hcrt2) since they are secreted from the hypothalamus and due to their significant amino acid homology with the member of the incretin family, the gut hormone secretin (glucagon/vasoactive intestinal polypeptide/secretin) [3,4]. Nowadays, both terms are used interchangeably in the research world and the literature. In this work, to avoid possible confusion, we use the term orexin (Orx).

Initially described as regulators of feeding and appetite behavior, subsequent research discovered that orexin or orexin–receptor deficiencies caused narcolepsy in different mammalian species, demonstrating that orexins are important in the regulation of the sleep and wakefulness states [5,6,7,8]. Soon after the discovery of orexins, a great number of studies have been conducted to elucidate their functions, providing evidence that orexin neurons have a projection pattern widely distributed to most parts of the central nervous system (CNS). These distributed projections allow orexins to influence several target regions and to be able to regulate a great range of physiological factors. These characteristics of orexins lead to their description as multitasking neuropeptides [9]. In fact, more recent research has been focused on the key role of orexins in a broad range of biological functions, such as the regulation of emotional states, energetic homeostasis, motor and autonomic functions, reward mechanisms, addiction, attention, the arousal system, and the sleep and wakefulness states [4,9,10,11,12,13,14,15,16]. The demonstration of the physiological implications of the orexin system are constantly growing, showing that orexins are a key player in the links between many different systems of our organism.

## 2. The Orexin/Hypocretin System

**Figure 1:**
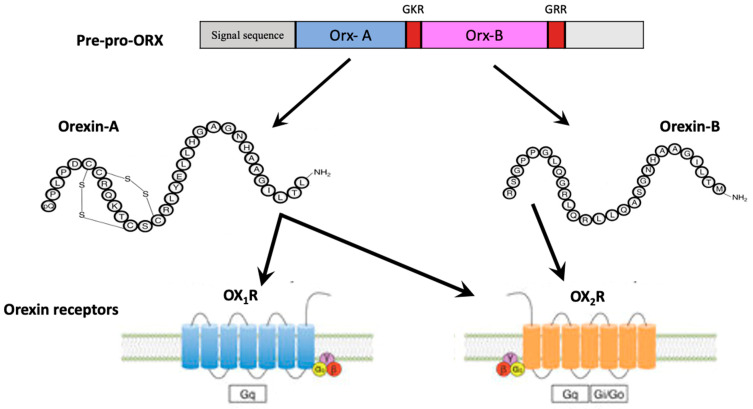
Orexin structure and receptors. Orx-A and Orx-B are derived from a common precursor peptide, pre-pro-orexin (pre-pro-Orx). The actions of orexins are mediated via two GCPRs, orexin-1 (OX_1_R) and orexin-2 (OX_2_R) receptors. OX_1_R exerts greater affinity for Orx-A, whereas OX_2_R is a non-selective receptor for both Orx-A and Orx-B.

In mammals, orexins are neuropeptides synthesized by a cluster of neurons within the lateral hypothalamic area (LHA), including the perifornical, posterior, lateral, and dorsomedial nuclei, producing excitatory effects on target neurons [4,5,6,7,8,9,10,11,12,13,14,15,16,17]. Orexins are synthesized starting from a common precursor, pre-pro-orexin, due to proteolytic processing. In humans, the gene encoding of pre-pro-orexin is located on chromosome 17, and its mRNA encodes for a 131-residue precursor peptide [18] (Figure 1). This precursor is processed through proteolytic cleavage, obtaining OrxA, a 33-amino-acid peptide with two intrachain disulfide bonds (C6–C12 and C7–C14), an amino(N)-terminal pyroglutamyl residue and carboxy (C)-terminal amidation, and OrxB, a linear 28-amino-acid peptide with C- terminal amidation [1,9,19]. The structures of OrxA and OrxB are highly conserved among all mammalian species, with higher amino acid homology for OrxB among mammalian species [9]. Furthermore, the C-terminal portion of OrxB is similar to that of OrxA, whereas the N-terminal portion presents more variability. The highly conserved structures of these peptides could be related to the importance of their functions [9]. Orexin functions are mediated by specific receptors, OX_1_R and OX_2_R, which are members of the GPCRs [9]. These receptors share 63.5% of amino acid homology. In humans, the OX_1_R gene is located on chromosome 1, and the OX_2_R gene is on chromosome 6 [9,19]. OX_2_R has been suggested as an ancestral form of an orexin receptor since it is present in all vertebrate genomes [9]. On the other hand, having been found only in mammals, OX_1_R, is considered an evolutionary product of duplication of the gene encoding OX_2_R [9]. This evolution could be related to the physiological roles that are more complex for OX_1_R. OX_1_R exerts greater affinity for OrxA than OrxB, and its activation increases the intracellular Ca^2+^ concentration, while OX_2_R binds both orexins with similar affinities and its activation could transmit signals via the G inhibitory protein class [20]. OXRs activate different G proteins, such as Gq, Gi/o, and Gs, demonstrating the complex intracellular signaling cascades activated by orexins [21]. Knowledge about neuronal orexin receptor signaling is limited since it is not clear which G proteins are the main signal transducers. Generally, the activation of orexin receptors leads to neuronal depolarization by K^+^ channel inhibition and Na^+^ influx due to the activation of Na^+^/Ca2^+^ exchangers and non-selective cation channels for which signaling cascades remain unclear [21,22]. OXRs are highly expressed in the CNS, with some regions that express both, and others, only one. Therefore, they exhibit different distribution patterns that support the different physiological roles of OX_1_R and OX_2_R and are greatly expressed on monoaminergic and cholinergic neurons in the brain stem and brain areas related to arousal, attention, stress, and reward [23,24,25,26] (see Table 1 for OX_1_R and OX_2_R brain expression).

**Figure 2:**
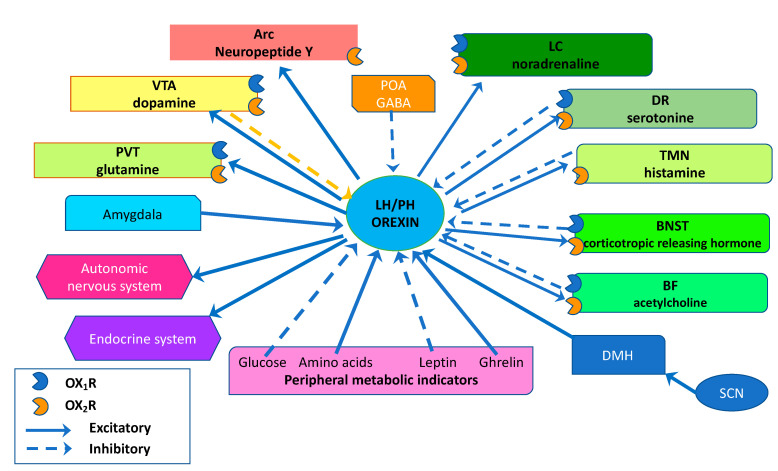
Schematic representation showing the main projections of orexin neurons and receptor expressions in the human brain. Orexin neurons from the lateral hypothalamic area (LHA) and posterior hypothalamus (PH) are anatomically well-placed to create links with several brain areas, regulating sleep and wakefulness states by sending projections to the entire CNS, mainly to the monoaminergic nuclei and cholinergic nuclei in the brain stem and hypothalamic regions, including the locus coeruleus (LC), basal forebrain (BF), tuberomammillary nucleus (TMN), and dorsal raphe nuclei (DR). The orexin system can also modulate the reward system, mainly through the stimulation of dopaminergic centers, such as the ventral tegmental area (VTA) and feeding behavior due to projections to the arcuate nucleus (Arc). Peripheral metabolic indicators, such as glucose, amino acids, ghrelin, and leptin levels, can influence orexin system activity to maintain energy homeostasis. Moreover, according to the arousal state, the orexin system regulates sympathetic outflow and the neuroendocrine system. Solid arrows indicate excitatory projections, and broken lines indicate inhibitory projections.

In humans, orexin neurons have been estimated to range between 50,000 and 80,000 neurons, localized in the hypothalamus, with fibers that widely project to several brain areas and all spinal cord levels. The densest projections extend to monoaminergic and cholinergic nuclei in the brain stem, BF, LC, DRN, TMN, and VTA [27,28] (Figure 2).

The major projections from Orx neurons related to the regulation of arousal and sleep/wakefulness are: intrahypothalamic innervation, monoaminergic arousal systems, cholinergic basal forebrain, and medial thalamus [4,5,6,7,8,9,10,11,12,13,14,15,16,17]. From Orx neurons, the densest extrahypothalamic projections are sent to the LC, where the co-release of orexins, as well as glutamate, stimulate noradrenergic (NE) neurons, demonstrating the important involvement of the orexin system in the modulation of sleep/wakefulness and the reward–addiction process [29,30,31,32]. Orexin neurons project to the VTA and the Orx–VTA neuronal circuitry is implicated in the modulation of reward–addiction, motivated behavior, and sleep [33]. Indeed, Orx neurons synapse on VTA dopaminergic (DA) neurons, exciting them to promote arousal, while VTA GABAergic neurons synapse on LH Orx neurons to inhibit them and induce NREM sleep [34,35]. Orexin neurons also project densely to the BF cholinergic neurons to drive wakefulness and to promote attention and motivated behavior [15,36,37,38,39,40]. Meanwhile, LH Orx neurons receive projections from BF glutamatergic and GABAergic neurons, and these neuronal circuitries may consolidate the wakefulness required for attention [40]. Orexin neurons also create bi-directional connections with DR serotonergic neurons that are important in the modulations of reward processing and sleep [41,42,43,44]. Orexinergic projections in the paraventricular thalamus (PVT), instead, are associated with the release of OrxA and OrxB and the co-release of dynorphin, which counteracts the excitatory effects of orexins on PVT neurons [45]. Orx–PVT interactions are involved in the modulation of sleep, reward–addiction, cognition, and stress [25].

Dense projections of orexin neurons that arrive at the paraventricular nucleus (PVN), and the Orx–PVN pathway are involved in addiction and sympathetic function [25]. Other widely orexin-innerved areas are the bed nucleus of the stria terminalis (BNST), where the Orx–BNST pathway plays a key role in arousal, sustained wakefulness, and brain homeostasis, and the tuberomammillary nucleus (TMN), where the Orx–TMN histaminergic bidirectional pathway is involved in wakefulness consolidation in appropriate circumstances [46,47,48,49]. Moreover, orexin projections to the ventral pallidum (VP) are involved in hedonic valence processing, while those to the insular cortex may be involved in orexin signal amplification during this processing [25]. Dense projections of orexin to the arcuate nucleus exert a crucial role in the modulation of feeding and body weight [50,51,52]. Therefore, due to all of its connections, some of which are bi-directional, the orexin system is crucial for regulating several activities that require arousal.

## 3. Sleep/Wakefulness States Regulation

The role of orexins in the regulation of the sleep and wakefulness states has been treated and discussed in many studies, starting with the discovery that orexin or Orx–receptor deficiencies cause narcolepsy in different mammalian species, demonstrating that orexins play a crucial role in the maintenance of wakefulness [2,5,6,7]. Narcolepsy is a neurological disease characterized by chronic and excessive daytime sleepiness, instability of sleep onset and REM periods, sleep paralysis and attack, and cataplexy [53]. For the identification of narcolepsy, it is not necessary that all the symptoms are present together and, at all ages, the diagnosis is possible via standard polysomnography [54]. The major causes of narcolepsy are a reduction or loss in the functions of orexinergic neurons or orexin receptors [36,55]. Generally, the maintenance of wakefulness and adequate arousal levels are needed for any purposeful behavior that requires elevated motivation. The systems involved in the regulation of wakefulness and arousal are tightly interconnected with the orexin system [56]. In fact, the integrative process of physiological, emotional, and environmental stimuli, both interoceptive and homeostatic, of the orexin neurons is a key role in promoting wakefulness and arousal in response to circadian rhythms, stress, emotions, and hunger [57]. The orexin system mediates arousal in response to food shortage and negative energy balance [58,59]. Thus, orexinergic signaling could result in increased wakefulness during acute fasting [12]. It has been highlighted that the electrical activity of orexin neurons is modulated by the energy status responding to hormonal and nutrient signals through neural pathways shared by feeding centers, such as the ARC and DMH [60]. Further, orexinergic feedback may directly interact with SCN control when excitatory metabolic stimuli require temporal flexibility [61].

The circadian timing system contributes to sleep regulation and energy homeostasis [62]. In mammals, the master circadian clock of the SCN integrates temporal information to control sleep and energy balance through its pathways. However, while the SCN is relatively insensitive to an altered metabolic status (e.g., prolonged fasting or mealtime constancy), the circadian system is able to adapt to changes according to food availability [62]. The circadian system plays a central role in the mechanisms regulating sleep and energy expenditure under conditions of metabolic stress. Animals reorganize many behavioral and physiological processes in response to constant and predictable food intake [63]. It has been shown that limiting food access to the light time phase induces an anticipatory increase in locomotor activity, body temperature, and circulating corticosterone in anticipation of the new feeding time [64]. This anticipatory behavior is termed food anticipatory activity (FAA), and it is independent of SCN control, mostly aligned with the prevailing light–dark (LD) cycle [65]. However, FAA exhibits the properties of an underlying circadian clock process and requires at least part of the canonical clock for its occurrence [66,67]. Importantly, the behavioral entrainment of restricting feeding and the development of FAA lead to a pronounced reorganization of locomotor activity and sleep patterns during the day and night. It has been suggested that food anticipation behavior and reorganization of physiological and behavioral patterns during restricted feeding experimental protocols are driven by one or more food entrainment oscillators (FEOs) [68].

This integration is possible due to the distribution innervation of orexinergic neurons to all brain regions known to promote wakefulness and arousal, including the BF, LC, DRN, TMN, and BNST [4,57,69]. 

### 3.1. Orexinergic Neurons and Basal Forebrain (BF) Projections

The bi-directional connections between orexin neurons and BF play important functions in the modulation of sleep and wakefulness states and attention. Orexins stimulate BF cholinergic and non-cholinergic neurons in response to salient stimuli to activate the cerebral cortex, drive wakefulness, and promote attention [15]. Mainly, Orx neurons synapse on BF cholinergic neurons, depolarizing them, and this pathway plays a critical role in wakefulness and attention modulation [38,39]. The injection of orexins to the BF stimulates wakefulness, decreases NREM sleep, and increases acetylcholine release in the somatosensory cortex, with greater stimulation with OrxA than OrxB [36,70]. These effects in the BF show a dose-dependent response [71]. Orexin neurons also synapse on BF glutamatergic neurons, exerting a physiological role in consolidated wakefulness, which is required for attention [40]. 

### 3.2. Orexinergic Neurons and Locus Coeruleus (LC) Projections

Orexin neurons densely project to LC noradrenergic (NE) neurons, modulating their activity via orexins or glutamate co-released by Orx neurons [29,30]. LC NE neurons stimulate the sleep-to-wake transition and show a specific fire pattern with a higher frequency during the wake state and a lower frequency during NREM sleep, while they are silent during REM sleep [72,73,74]. While Orx and LC NE neuron stimulation increases the possibility of sleep-to-wake transitions, the administration of OrxA (but not OrxB) to the LC stimulates wakefulness and dampens REM sleep [75,76]. Furthermore, orexin neurons also innervate LC neurons, both NE and non-NE, that project to the medial prefrontal cortex (mPFC) to sustain wakefulness. LC neuron activity in the mPFC may be mediated by OrxA, showing its involvement in cortical activation, sleep/wakefulness state stabilization, and arousal [77]. All these studies support the involvement of the orexin system and the Orx–LC pathway in sleep/wake regulation. 

### 3.3. Orexinergic Neurons and Dorsal Raphe Nuclei (DRN) Projections

The Orx–DR neuronal circuitry is involved in sleep modulation and reward processing through orexinergic projections to the DR serotoninergic neurons. DR serotoninergic neurons discharge tonically with a low frequency during wakefulness and exposure to reward stimuli, but their activity is driven by the excitatory orexinergic input [43,78,79,80]. Other neuronal circuitries in which orexins neurons play an important role in sleep/wakefulness state regulation are the TMN histaminergic neurons and the BNST GABAergic neurons. 

### 3.4. Orexinergic Neurons and Tuberomammillary Nucleus (TMN) Projections

Specifically, TMN histaminergic neurons fire during wakefulness. These neurons are reciprocally connected to orexin neurons and are innervated by GABAergic neurons [47,48,49]. Orexin neurons directly stimulate TMN histaminergic neurons via Orxs and glutamate or indirectly via GABAergic neuron suppression via dynorphin and subsequent TMN histaminergic neuron disinhibition [49,80,81,82]. Li and de Lecea (2020) showed that the activity of the Orx–TMN neuronal circuitry may lead to wakefulness consolidation [25]. 

### 3.5. Orexinergic Neurons and Bed Nucleus of Stria Terminalis (BNST) Projections

BNST is considered the center of “valence surveillance” to monitor both physical and social contexts, and shows intensive Orx neuron projections [4,83]. This monitoring requires high arousal levels in which the orexin system is a key player. In fact, the BNST contains a population of GABAergic neurons that release also corticotropin-releasing hormone (CRH), which activates orexin neurons. Kodani and colleagues demonstrated that the prolonged stimulation of these BNST GABAergic neurons excited orexin neurons to sustain wakefulness mostly through NREM sleep inhibition [46]. 

All these neuronal pathways demonstrate the complex key role of the orexin system in sleep/wakefulness state regulation, but given the high number of connections of this system, future research and novel data might elucidate the different orexinergic activity of this regulation, both direct and indirect.

## 4. Feeding Behavior, Energy Homeostasis, and Obesity

Eating behaviors are modulated by several hormones and neuropeptides. Among them, orexins play a meaningful role [84,85,86,87,88,89]. Several studies have focused on the role of orexins in feeding behavior and the regulation of food intake, showing that orexin blockade or the genetic or toxic ablation of orexin neurons [11,90,91,92,93] leads to hypophagia. In contrast, central but not peripheral administration (e.g., ICV injections or intranasal administration of OrxA) increased food intake [94,95,96,97,98,99,100,101]. Orexin-modulated effects on feeding behavior, energy expenditure, and obesity are reported below.

### 4.1. Orexin and Feeding Behavior

Microinjections of OrxA into the LH and perifornical hypothalamus, DMH, PVN, mPOA, and ARC were shown to increase food intake [51]. This effect was also obtained with OrxA microinjections into extra-hypothalamic sites, such as the NAC, nucleus incertus, and basomedial amygdala [102]. In addition, experiments with OX_1_R antagonists, such as SB-334867, have demonstrated reduced food intake in rats and involvement of orexins in feeding stimulation. In this regard, preclinical models of binge eating suggest treatment with OX_1_R antagonists as a therapeutic approach to reduce binge-like eating behavior [103], a hallmark of bulimia nervosa [104]. However, the role of OrxB in feeding behavior seems to be liminal and unclear [102] and needs further investigation. A number of studies have reported the role of orexins in hedonic or reward-based (non-homeostatic) eating. Orexin does not simply increase consumption indiscriminately; the salience or the palatability of food rewards is the main factor driving the behavior [105]. This includes the overconsumption of highly palatable food that is rich in fat and sugar in the absence of caloric need [102,106,107]. In fact, projections between the prefrontal area and hypothalamic circuits appear to be relevant for the modulation of hunger and satiety signals [108,109]. The preference for agreeable food is related to a significant increase in orexin gene expression in the perifornical area [110] and the double expression of orexin and Fos (a marker of neuronal activation) in the lateral hypothalamus [13]. Further, orexin cells produced significantly higher calcium signals when mice approached a chocolate pellet, suggesting that the salience/palatability of food could increase orexin neuron activity to promote appetitive behavior [111]. Orexins are orexigenic neuropeptides with a distinct effect since they increase food intake, similar to other orexigenic peptides, but they also increase energy expenditure, in contrast to other orexigenic peptides that generally decrease it to preserve energy in case of a lack of food [9,112]. The increase in feeding behavior observed after ICV OrxA injection may be due to the action on the LH, which contains neurons whose activity is modulated by glucose concentration [54]. The orexin system senses the physiological change in glucose levels associated with meals and, in turn, modulates energy balance. Orexin neurons are excited by low extracellular glucose concentrations, decreased leptin levels, and increased ghrelin levels, and therefore, when a negative energy balance is present [42,113,114]. Leptin inhibits orexin neurons that receive projections by neurons in the arcuate nucleus, the main sensor for leptin levels in plasma [42,115]. Referring to ghrelin, an ICV injection of ghrelin in goldfish diencephalon led to an increase in pre-pro-orexin mRNA expression and an increase in food intake, which, in turn, was inhibited with OXR antagonist administration [116]. Increased extracellular nonessential amino acid levels also influence orexin neuron activity, depolarizing them; this may be a physiological response to prolonged starvation, in which the increase in extracellular amino acid levels is a consequence of protein breakdown for fuel [117]. These studies support the role of the orexin system as a sensor for energy balance indicators and an animal’s metabolic and nutritional status to integrate all the stimuli, maintain a persistent wake state, and promote alertness to food-seeking behavior when needed.

### 4.2. Orexin and Energy Expenditure

Orexins can regulate different physiological functions and exert a critical role in energy balance via their distributed projections to several areas of the CNS, including critical areas for the regulation of physical activity, such as the DRN, LC, and substantia nigra [118,119].

The orexin-induced increase in energy expenditure could be related to an increased state of wakefulness and vigilance, the resulting increase in locomotor activity, and the activation of sympathetic tone [112]. In contrast, orexin deficiency is associated with decreased sympathetic tone (and a relative decrease in wakefulness and locomotor activity), resulting in decreased energy expenditure [12,120,121]. In addition, decreased energy expenditure could increase body weight, as observed in narcoleptic subjects, despite eating less food [11,122]. Therefore, increased orexin activity is associated with decreased body weight [123]. Indeed, in the occurrence of negative energy balance (e.g., induced by reduced food availability), mammals activate a number of physiological responses that alter energy expenditure and the level of behavioral arousal aimed at increasing food-seeking ability. In this respect, several studies have demonstrated that orexin neuron activity is influenced by some energy balance indicators, such as glucose, triglyceride, and amino acid concentrations, as well as leptin and ghrelin levels [12,113,117,124].

It is well known that physical activity improves general health [125,126,127,128], prevents metabolic disease, and resists obesity [119,129,130]. The main contributors to human susceptibility and variability against weight gain are spontaneous physical activity (SPA), which refers to any physical activity not due to voluntary exercise, and “non-exercise-activity thermogenesis” (NEAT), the thermogenesis associated with SPA and embracing all the energies employed in moving and standing [130,131]. In humans, nearly 30% of daily energy expenditure results from SPA and NEAT, and the ability to increase them could be a protective response against obesity [118,130]. SPA and NEAT are important for energy homeostasis, and several peptides have been proposed as their modulators, including orexins [130,131]. Indeed, the orexin system regulates energy expenditure by increasing walking, and consequently, SPA, and reducing sedentary time [130,131].

### 4.3. Orexin and Obesity

In recent decades, obesity has assumed an alarming clinical and social prominence. Excess body weight is linked to several chronic diseases and reduces both the quality of life and life expectancy [132,133]. Murine studies have demonstrated the existence of obesity-resistant (OR) model rats that have high intrinsic SPA and enhanced orexin-induced SPA [134,135] and high-activity (HA) model rats (identified by their endogenous SPA) that have high resistance to obesity following being fed with a high-energy diet [136]. These data confirm that the resistance to diet-induced obesity is highly influenced by an individual’s propensity for SPA and support that the orexin system regulates energy expenditure, increasing SPA and reducing sedentary time [134,135,137]. 

Furthermore, in obese and overweight subjects, higher plasma OrxA levels are associated with a moderately active lifestyle [138]. In contrast, in diet-induced obese animals, both the CNS and peripheral tissues showed low orexin levels, while in obese humans, low orexin concentrations in adipose tissue were found [139]. Acting as metabolic sensors, orexin neurons are inhibited by high glucose and leptin plasma levels, and a decrease in orexin activity might promote obesity, decreasing SPA and energy expenditure [9,31,140]. A higher orexinergic tone is considered an endogenous factor that predicts physical activity and, in turn, improves energy expenditure and body weight. Subjects who are physically active show a higher OrxA plasma level [138]. 

Conversely, obese people and obese-induced animals have low physical activity and plasma orexin levels. In these subjects, weight loss leads to enhanced plasma orexin levels as well as improved sleep quality [119,141]. Furthermore, physical exercise increases plasma OrxA levels, which stimulates the sympathetic nervous system and energy expenditure, and OrxA seems to be able to promote thermogenesis during physical activity [118,130,142,143,144]. To support the role of orexins in obesity resistance, several animal studies have demonstrated that OrxA injections into several cerebral areas, such as the rostral LH, paraventricular nucleus, TMN, LC, DRN, nucleus accumbens, and substantia nigra, improve SPA and NEAT [132,145,146,147,148,149,150,151,152], while repeated injections of OrxA into LH are associated with reduced fat mass [153]. Orexins also exert a sympathoexcitatory effect, as demonstrated by increased blood pressure and heart rate, increased sympathetic outflow to brown adipose tissue (BAT), and increased plasma epinephrine and noradrenaline release [141,154,155,156]. The sympathetic outflow to BAT may be a cause of the increase in NEAT due to orexin [157]. Some studies on animals have suggested that obesity resistance is related to the overexpression of pre-pro-orexin in the LH and greater OrxA sensitivity, mainly in the rostral LH, to increase SPA [123,135,136]. 

The orexin–DRN serotonin neuronal circuitry also promotes physical activity and energy expenditure, integrating metabolic signals and activating specific behaviors following energy needs [118]. The complex orexin system network is involved in feeding behavior and energy balance, and in food-deprived animals or dieting humans, orexin mRNA and Orx receptor expressions are increased [113,117,158].

Thus, considering these data, orexin neuron activity and OrxA sensitivity might be new therapeutic approaches to resisting obesity, increasing SPA, energy expenditure, and brown adipose tissue thermogenesis, with positive effects on general health.

## 5. Reward System and Addiction 

Recent studies have focused on the role of the orexin system in reward and motivation, suggesting that this system coordinates motivational activation in several behavioral conditions [159]. Indeed, orexin system plasticity seems to be a neuronal signature involved in alcohol use and drug addiction [10,160,161,162]. Orexins’ involvement in addiction might be related to their ability to stimulate synaptic function in the main reward areas of the brain. The projections of the orexin system to the NAC, VTA, LC, and DRN represent interactions that well-localize the orexin system as a mediator of the effects of drugs and so forth [32,43,162,163]. Orexinergic projections to the LC and VTA create a stronger association between arousal and reward-seeking [31]. Following drug-associated stimuli, orexin neurons are excited to activate motivated behavior via outputs to reward centers, including the VTA, in which the major clusters of DA neurons are present [164]. In the VTA, orexin neurons directly stimulate DA neurons due to their direct synapses but also indirectly via glutamate. Indeed, orexin neurons presynaptically promote the release of glutamate by glutamatergic neurons and postsynaptically facilitate the synaptic translocation of glutamate receptors on DA neurons [165]. These orexinergic activities enhance glutamate release onto DA neurons and, at the same time, enhance the probability of the firing of DA cells, facilitating glutamatergic signaling. This plasticity increases the activity of the neuronal circuitry in response to the next expositions to drug-associated stimuli, leading to hypermotivated, drug-seeking behaviors [10]. Indeed, drug exposure increased the stimulation of VTA DA neurons, and the use of OX_1_R antagonist in the VTA inhibited the locomotor-enhancing effects of repeated cocaine exposure [165]. These observations suggest that orexins play a central role in motivated behaviors and the drug-induced upregulation of DA neurons. 

Furthermore, in morphine-treated rats, OrxA enhanced the suppressive effect of naloxone on the GABAergic inhibitory postsynaptic currents (IPSCs) of LC neurons, demonstrating its key role in regulating LC GABAergic neurons [32]. Therefore, the Orx–LC pathway exerts a critical role in addiction processing. Generally, orexins are involved in increased arousal, wake states, and motivation to create appropriate goal-directed behaviors. In addicted states, when environmental stimuli related to drug availability are present, the orexin system discharges, stimulating all the neuronal circuitries to activate appropriate behaviors to obtain the drug and then regulating the motivated drug-seeking behaviors that characterize addiction [10]. Drug-seeking behaviors are increased after orexin stimulation, while OX_1_R antagonists decrease drug-seeking behavior and relapse in model animals [163]. Specifically, selective OX_1_R antagonists (SORA-1s) reduce various drug-seeking behaviors, mainly those due to drug stimuli, including relapse, without sedative effects [10,166]. Recently researchers have focused their attention on the orexin system as a therapeutic approach to clinically manage substance-use disorders. In this light, the National Institute on Drug Abuse (NIDA) added orexin receptor antagonists as a target for new medications for the opioid crisis [167]. Decreases in drug-seeking behavior and alcohol consumption were also observed in animals highly motivated for alcohol consumption, and this result might be related to the effects of norepinephrine. The use of OXR antagonists has been suggested to treat addiction-induced anxiety and sleep disturbances in alcohol abuse patients to prevent relapse, enhance sleep, and prolong the length of abstinence [159,166,168]. Taken together, these data support the key role of orexin in the reward-system mechanisms of drug addiction and as a potential target for promising pharmacotherapies to treat addictions, manage cravings, and reduce relapse in substance-use disorders.

## 6. Ageing and Neurogenesis

Both aging and neurodegeneration have shown variations in neuronal numbers and functions following a decrease in the functional capacity of the subject [169,170,171]. Few data are available from post-mortem clinical studies in humans on orexin neurons in neurodegeneration cases. The hypotheses regarding the involvement of the dysregulation of the orexinergic system and/or orexin receptors in the pathogenesis of neurodegenerative diseases, such as Parkinson’s disease (PD) and Alzheimer’s disease (AD), are supported by recent evidence [172]. Fronczek et al. (2012) demonstrated a significant decrease of 40% in hypothalamic orexin neurons in advanced Alzheimer’s disease patients, associated with low orexin ventricular cerebrospinal fluid (CSF) concentrations [173]. A decrease of about 40% in orexin neurons was also observed in the prefrontal cortex of patients with PD, with a halving in the total number of orexin neurons [174]. In mice models of PD, Stanojlovic and colleagues (2021) pointed out the important role of Orx neurons in PD-associated pathology, demonstrating a reduction in orexin neurons and the implication of orexins in the variations of hypothalamic-regulated physiological functions related to Parkinson’s disease [175]. A decrease in Orx neuron numbers related to age was also observed in the LCs of macaques and cats, as well as in the LH of rats [18,176,177,178]. Furthermore, data from animal studies support the role of the orexin system as a hippocampal neurogenesis enhancer, influencing memory and learning processes. Zhao et al. (2014) demonstrated increased neurogenesis in the dentate gyrus following OrxA ICV injection in rats [179]. The orexin system might also be involved in the ability to discriminate familiar from new conspecifics and remember them—the so-called social memory. Indeed, orexin/ataxin-3-transgenic animal models (animals that show orexin neuron degeneration due to specific expressions of the ataxin-3 toxic transgene in orexin-containing neurons) showed deficits in long-term social memory, and further, a decrease in these deficits and enhancement in synaptic plasticity in the hippocampus was obtained following nasal OrxA administration [180]. 

Notably, there are also relationships among physical activity, the brain, and orexins. Indeed, physical activity increases plasma orexin levels, and at the same time, has been demonstrated as an efficient tool to enhance hippocampal neurogenesis and function, improve cognition, and regulate mood [142,143,181,182,183,184]. Several factors might contribute to these beneficial effects, such as increased vascularization and the upregulation of growth factors, as well as OrxA, which enhances both hippocampal neurogenesis and its functions. This bi-directional relationship between physical activity and orexins might be an important resource to prevent and treat aging-related cognitive decline and some neurodegenerative diseases.

## 7. Conclusions

In the last several years, new research, data, and information on the orexin system have been useful to revise, increase, and clarify our current understanding of this system. All the connections and the neuronal circuitries of the orexin system explain the multitasking role of orexin neurons in the regulation of wakefulness, arousal, the reward/addiction system, SPA, as well as energy homeostasis. Therefore, the orexin system represents a key system for a healthy life, regulating not only physiological but also metabolic functions. Over the last few years, the orexin system has progressed to being a promising target for new medications and therapeutical approaches, along with physical activity, such as to resist obesity, increase SPA and energy expenditure, treat addiction, and prevent and treat aging-related cognitive decline. However, given the high number of connections of this system, future research and novel data might elucidate both direct and indirect activities of the orexin system and how it regulates attention, energy homeostasis, emotions, feeding behavior, and the reward system.

## Figures and Tables

**Table 1:** Brain expression sites for OX_1_R and OX_2_R [18,23,24,25,26].

OX_1_R Expression Site	OX_2_R Expression Site
Nucleus of the Solitary Tract (NTS)	Nucleus of the Solitary Tract (NTS)
Pedunculopontine/Latero-Dorsal Tegmental Nucleus (PPT/LDT)	Arcuate Nucleus (ARC)
Locus Coeruleus (LC)	Pedunculopontine/Latero-Dorsal Tegmental Nucleus (PPT/LDT)
Ventral Tegmental Area (VTA)	Locus Coeruleus (LC)
Dorsal Raphe Nucleus (DRN)	Ventral Tegmental Area (VTA)
Anterior Hypothalamus	Dorsal Raphe Nucleus (DRN)
Bed Nucleus of the Stria Terminalis (BNST)	Paraventricular Thalamus (PVT)
Basal Forebrain (BF)	Paraventricular Nucleus (PVN)
Paraventricular Thalamus (PVT)	Preoptic Area (POA)
Paraventricular Nucleus (PVN)	Lateral Hypothalamus (LH)
Preoptic Area (POA)	Basal Forebrain (BF)
Hippocampus (CA1 And CA2)	Bed Nucleus of the Stria Terminalis (BNST)
Dentate Gyrus (DG)	Dorsomedial Hypothalamic Nucleus (DMH)
Amygdala	Tuberomammillary Nucleus (TMN)
Ventral Pallidum (VP)	Hippocampus (CA3)
Olfactory Bulb (OB)	Dentate Gyrus (DG)
Prefrontal and Infralimbic Cortex (IL)	Amygdala
Insular Cortex (IC)	Nucleus Accumbens (NAC)
	Lateral Septum (LS)
	Medial Septum (MS)
	Anterior Commissure (AC)

## Data Availability

Not applicable.

## Abbreviations

AC	anterior commissure
Arc	arcuate nucleus
BAT	brown adipose tissue
BF	basal forebrain
BNST	bed nucleus of the stria terminalis
CNS	central nervous system
CSF	cerebrospinal fluid
DG	dentate gyrus
DMH	dorsomedial hypothalamic nucleus
DRN	dorsal raphe nucleus
GABA	gamma-aminobutyric acid
GPCR	G protein-coupled receptor
HA	high activity
IC	insular cortex
ICV	intracerebroventricular
LA	low activity
LC	locus coeruleus
LHA	lateral hypothalamic area
LS	lateral septum
MS	medial septum
NAc	nucleus accumbens
NEAT	non-exercise induced thermogenesis
NREM	non-rapid eye movement
NTS	nucleus of the solitary tract
OB	olfactory bulb
OP	obesity prone
OR	obesity resistant
OrxA	Orexin-A
OrxB	orexin-B
PD	Parkinson’s disease
POA	preoptic area
PPT/LDT	pedunculopontine and latero-dorsal tegmental nucleus
PVN	paraventricular nucleus
PVT	paraventricular thalamus
REM	rapid eye movement
SPA	spontaneous physical activity
TMN	tuberomammillary nucleus
VP	ventral pallidum
VTA	ventral tegmental area
